# System of Surgery

**Published:** 1836-07

**Authors:** 


Art. IX.—*System der Chirurgie.* Von P. F. Von Walther, Doctor der Philosophie, Medicin und Chirurgie, Offentlich. Ordentl. Lehrer in der Medic. Facultät der Ludwig Maximilians Universität, &c. &c. &c. Berlin, 1833. Erster Band. Seite 418.

*System of Surgery*, by P. F. Von Walther. 1st vol. pp. 418. Berlin, 1833.

This production of Von Walther, a professor and teacher of clinical surgery at Bamberg, Landshut, Bonn, and now at Munich, for upwards of thirty years, well known too by various surgical and physiological productions, more particularly the Journal of Surgery and Ophthalmology, edited since 1820, in conjunction with Graefe, will hereafter give the means of estimating the present state of the theory and practice of surgery as taught in the schools of Germany. The present volume is the first of a very comprehensive system, in which surgical diseases are to be treated of as they occur in various organs and regions of the body. At the same time, it is complete in itself; inasmuch as it embraces a consideration of general surgical pathology and therapeutics, presenting, in this respect, the same relation to special surgery, as a treatise on general to descriptive anatomy.

Professor Von Walther spares no trouble in proving his sense of the importance of his subject matter, by demonstrating that the distinction between physic and surgery in point of truly scientific value is factitious, having its origin neither in physiology nor in general pathology and therapeutics, but arising solely from circumstances connected either with peculiarities in practice or the artificial states of society. To use his own language :—

"The various forms in which disease presents itself constitute an immense series, throughout the whole extent of which there is no discontinuity, the amount of diversity which eludes observation between individual members of the series only becoming discernible when we contrast with each other its extremes.

"Thus, on the one hand, there are diseases, the essential character of which consists simply in injuries or imperfections succeeded by consequences purely physical and mechanical in their nature and effects. On the other, we meet with maladies which consist primarily in modifications of the vital powers,—in functional disturbances without any preceding physical abnormal changes, and which, originating in the very sources of vitality, speedily extend their influence over entire systems of organs or even the œconomy at large.

"It is on this apparent opposition that rests the distinction of diseases into surgical and medical, being the consequence of attaching undue importance to one set of circumstances to the exclusion of others, or even of an over estimate of the relative value of points which are still more subordinate." (P. 3.)

"Medicine and surgery are neither independent of the other in nature or in practice, the separation established between them being purely artificial; each in its present condition comprises a portion of the doctrines of pathology and practice, which are only to be rendered complete by the junction and intimate union of both." (P. 11.)

This imperfect and fragmentary state of surgery, as viewed in an isolated shape, affords a ready and sufficient explanation of the difficulties that present themselves in the way of all endeavours to arrive at a natural, and at the same time useful, classification of the objects which it embraces.

That such difficulties have been felt by surgical writers is sufficiently evinced by the extent of the differences displayed in this particular; which may, however, be referred to the greater or less predominance of one or other of two leading objects;—on the one hand, the desire to attain a completely natural or logical arrangement,—on the other, the sacrifice of all such views, to the great end of simplicity and practical utility.

Thus, adverting only to the writers of modern times, some, as Richter, Sabatier, and perhaps without exception all English authors, have classed surgical maladies according to the regions of the body affected; with the necessary addition of some precursory considerations on those more general, which are common to all parts of the system. Others, on the contrary, have adopted modes of arrangement, varying according as they are based on the presumed natural relations of different forms of disease, (Richerand, Chelius;) or, as some single circumstance or set of circumstances is assumed as the key to a more artificial distribution (Callisen, Boyer).

The method of Von Walther is of the latter kind, all surgical affections being divided among five classes arbitrarily assumed, viz.

1. Inflammation, with its terminations and results. (*Phlogosis.*)
2. Wounds, solution of continuity. (*Trauma.*)
3. Changes of situation. (*Ectopia.*)
4. Defects in form, mechanically producing disturbance of function and deformity. (*Pseudomorphia.*)
5. Foreign bodies, whether penetrating from without, or originating within the body. (*Allenthesis.*)

"The second, third, and fourth of these classes belong without dispute to surgery, and constitute its mechanical portion; whilst the first and fifth may be viewed as debateable ground, and have for their object what is called medical surgery." (P. 13.)

As in most other instances where the attempt is made to classify a very extensive collection of very diversified objects, the success of the endeavour is but partial, and leaves much room for criticism. Setting aside, however, difficulties that may be considered inevitable, we cannot but think that in some parts at least, Professor Walther's arrangement, at the same time that it wants the compensating quality of utility, needlessly sets at defiance all consideration of the true relations of the objects included in it.

This remark is not applicable to the first three classes, which are indeed at the same time so rational and so practical that it would be difficult to amend them. But in the fourth we find, not merely arranged together, but indiscriminately intermixed, affections the most dissimilar in their origin and nature.

For example, in the fourth class (Pseudomorphia,) we find included, on the one hand, various forms of congenital malformation, e. g. fissures in the middle line, atresia, synechia, excess and defect of parts;—and, on the other, aneurism, varix, distortions, and similar affections, which are to be viewed, not so much in the light of independent and clearly defined maladies, as of remote and by no means essential consequences of pathological changes occurring subsequent to birth, and the due appreciation of which forms the only satisfactory basis of classification.

The same neglect of the actual relations offered by the objects ranked together is not less obvious in the fifth class (Allenthesis;) in which, an agreement in the solitary fact of want of conformity to the original composition of the body forms the only ground for embracing under one head a long list of diseases confusedly and disadvantageously mixed together. In this latter instance, too, the error becomes the more striking as it evinces the neglect of grounds of subdivision resting on essential differences, and affording the opportunity of drawing general conclusions closely connected with practical purposes. Thus, foreign bodies penetrating from without, in conjunction with retained secretions and calculous concretions, might legitimately form one subdivision of the class; whilst another should be constituted by new formations (Pseudoplasma), which again are naturally divisible into, 1. Modifications of textures and organs previously existing, e.g. sarcoma, clavus, condyloma, cysts.—2. Deposits of natural textures in situations where their occurrence is abnormal, e.g. lipoma, steatoma, exostosis, and ossification.—3. Accidental productions, consisting of peculiar textures not analogous to the original composition of any part of the animal frame, e.g. polypus, scirrhus with carcinoma, and fungus, including encephaloma, neuroma, and melanosis.

Nor must it be supposed that criticism, such as we have ventured to institute upon these points, bears solely upon matters of theory, which might with indifference, as regards all useful ends, be settled in many different ways. For if, to use Von Walther's own language, "the surest mode of at once attaining brevity and correctness, whilst all needless repetition is avoided, consists in arranging each object to be examined in a suitable position with regard to every other,"—it is obvious, that the intention will be defeated by adopting a system that precludes the possibility of arriving at such general views of a mass of objects possessing natural connexions with each other, as would permit an easy and unstrained passage to an examination of those characteristic particularities by which the individuals differ from each other.

In the preceding remarks on Professor Von Walther's treatise it will be seen that we have abstained from any attempt at giving an analytical account of its contents, and chiefly for the reason that, consisting of a general and elementary exposition of the principles of surgical science, it is not of a kind to require or justify such a mode of proceeding. The spirit in which it is executed still farther confirms us as to the propriety of this course: the work is strictly dogmatic, with an almost utter exclusion of any attempt at entering upon discussion of unsettled questions, or estimating the relative value of contending opinions on points of theory or practice. We will not venture to decide whether this be a recommendation or otherwise; but in all other particulars there can be no hesitation in giving a favorable opinion. The language is clear, precise, and remarkably free from the parenthetical diffuseness of style which so frequently forms a heavy drawback on the merits of German scientific literature. By abstaining from a common practice among his countrymen, which consists in interweaving with practical every-day matters a certain vague and misty speculative philosophy, Von Walther succeeds in escaping the obscurity and want of reality which so often fatigue and disgust the English reader. Just enough, too, of notice is bestowed upon the literature of the science, by indicating to the student the best sources of information in general, and by quoting the latest and surest authorities on individual points, instead of burthening attention by a pedantic display of learning savouring more of vanity or trivial curiosity than of a sincere wish to accomplish a professed object.

We can therefore strongly recommend this work to such of our readers as are desirous of extending their acquaintance with the professional literature of Germany, and anticipate with gratification the more favorable opportunities which the succeeding volumes are likely to afford of comparing the condition of surgery among ourselves with that which it presents among Von Walther's countrymen.

Since the above notice was written we have been informed that Von Walther's work will ere long appear in an English dress, its translation having been undertaken some time since by a gentleman every way qualified to do it justice. We congratulate the English student on this circumstance, as we are certain the work will be found a valuable addition to scientific surgery, and tend to counteract the empirical taste which circumstances have for so many years tended to foster in the minds of surgical students in this country.

